# Nodal is involved in chemoresistance of renal cell carcinoma cells via regulation of ABCB1

**DOI:** 10.7150/jca.52092

**Published:** 2021-02-02

**Authors:** Xingwang Zhu, Dongwei Xue, Jia Liu, Fengming Dong, Yongzhi Li, Yili Liu

**Affiliations:** Department of Urology, The Fourth Affiliated Hospital of China Medical University, Shenyang, 110032, China.

**Keywords:** RCC, Nodal, P-gp, transcription, p65

## Abstract

Renal cell carcinoma (RCC) is the third most frequent malignancy within urological oncology. Understanding mechanisms of chemoresistance in RCC cell is important for therapy and drug development. We established cisplatin (CDDP) resistant RCC cells by treating cells with increasing concentrations of CDDP. Nodal, an important embryonic morphogen, was increased in RCC/CDDP cells. Targeted inhibition of Nodal via its siRNA or neutralization antibody restored sensitivity of RCC resistant cells to CDDP treatment. It was due to that si-Nodal can decrease expression of P-glycoprotein (P-gp, encoded by ABCB1), one important ATP-binding cassette (ABC) membrane transporter for drug efflux. si-Nodal can decrease the transcription and promoter activity of ABCB1. Mechanistically, si-Nodal can decrease the phosphorylation of p65, which can bind to the promoter of ABCB1 and then trigger its transcription. Further, CDDP treatment decreased the expression of Nodal in culture medium of RCC cells. Collectively, we found that Nodal can regulate chemoresistance of RCC cells via regulating transcription of ABCB1.

## Introduction

Renal cell carcinoma (RCC), which is also named as renal adenocarcinoma, accounts for about 90-95% of tumors in human kidney 1. Clear-cell RCC (ccRCC) is the most common type of RCC 2. Surgical is the mainstay of treatment for localized or early-stage RCC 3. Radiotherapy and chemotherapy can offer favorable prognosis for RCC patients at early stage 4. Immunotherapy and targeted therapy can also improve therapy efficiency 5. Unfortunately, some patients will develop chemoresistance after therapy for several months 6, which is a major obstacle in successful treatment in RCC. The molecular mechanism involved in chemoresistatnce of RCC has not been clarified yet, it is urgent to investigate the underlying mechanisms to improve therapy efficiency for RCC treatment.

Although multiple mechanisms are involved in chemoresistance, over expression of P-glycoprotein (P-gp, encoded by ABCB1) is considered as one of the most important mechanism for cancer chemoresistance 7, 8. P-gp can mediate the efflux of cytotoxic drugs to decrease their intracellular concentrations 9. The expression of P-gp has been widely detected in RCC tissues 10, 11. Further, RCC patients with none or few P-gp positive tumor cells had longer progression free survival than that of higher P-gp patients 12. Increased levels of P-gp were also observed in metastasized RCC patients compared to early stage patients 13. High frequency of tumors expressing P-gp suggests that they may be important contributors to the chemoresistance of RCC cells 14. In addition, many other family members such as ABCC1 (also named MRP1), ABCC2 (MRP2), ABCC3, and ABCG2 (BCRP) can also regulate RCC chemoresistance. Various transcription factors such as c-Jun, c-Fos, NF-κB/p65, and Sp1 can bind to the promoter of ABCB1 to increase its transcription 15, 16. Fox example, NF-κB/p65 can bind to -167 and -158 of the *ABCB1* promoter to activate its transcription in liver cancer cells 17. Since factors regulating transcription of ABCB1 might be cell line dependent, the illustration about factors regulating expression of these transporters is important to overcome chemoresistance of RCC.

Cytokines are reported to be critical for RCC progression 18. As one member of transforming growth factor β (TGF-β) superfamily, Nodal can re-express in various cancers, further, it is linked to aggressiveness in numerous cancer types 19. Nodal was expressed in human metastatic tumors to induce cell invasion, tumorigenicity, and chemoresistance 20. Nodal/Activin signals in cancer stem cells can promote the chemoresistance of pancreatic cancer 21. Nodal can contribute to dacarbazine resistance and maintain stemness in melanoma cells 22. As to RCC, it has been reported that Nodal can activate Smad and ERK1/2 signals to increase cell proliferation 23. However, the role of Nodal in chemoresistance of RCC has not been illustrated.

The present study established cisplatin (CDDP) resistant RCC GRC-1 and ACHN cells and observed that Nodal was increased in RCC chemoresistant cells. Targeted inhibition of Nodal can restore CDDP sensitivity of RCC cells, which was attributed to that Nodal can increase the transcription of ABCB1 via activation of NF-κB/p65 signals.

## Materials and methods

### Reagents

All chemicals were purchased from Sigma Chemical Co. (St. Louis, MO, USA) unless otherwise noted. Both neutralization antibody (sc-81953) and recombinant Nodal (rNodal) were purchased from Santa Cruz Biotechnology (Santa Cruz, CA, USA). The horseradish peroxidase (HRP)-conjugated secondary antibody was purchased from Cell Signaling Technology (Beverly, MA, USA). The inhibitors were purchased from Selleck, China (Shanghai, China). The compounds were solubilized in dimethyl sulfoxide (DMSO) with the final concentration less than 0.5% (v/v).

### Cell culture and establish of CDDP resistant cells

Human RCC GRC-1 and ACHN were purchased from the Cell Bank the Chinese Academy of Sciences (Shanghai) and cultured in Dulbecco's modified Eagle's medium (DMEM) supplemented with 10% fetal calf serum (FBS), 1% (v/v) penicillin, and 100 µg/mL streptomycin at 37 °C in a 5% CO_2_ atmosphere. To establish of CDDP resistant cells, both GRC-1 and ACHN cells were exposed to increasing concentrations of CDDP (0.01, 0.02, 0.05, 0.1, 0.2, 0.5 and 1 μM in normal medium) according to the recent study 14. After incubated for 6 months, CDDP resistant cells were generated and maintained in medium in the presence of 0.5 μM CDDP. The resistant cells were named as GRC-1/CDDP and ACHN/CDDP, respectively.

### Cell viability assay

The cell viability was measured by use of the MTT assay according to the previous study 24. Cells were cultured in 96-well plate with the density of 1 × 10^4^ cells per well. After incubated with CDDP for the indicated time periods, cells were incubated with 3-(4,5-dimethylthiazol-2-yl)-2,5-diphenyl-tetrazolium bromide (MTT) for 4 h at 37 °C. Then DMSO was used to dissolve the formazan crystals. The absorbance value at 490 nm was measured.

### Quantitative real-time PCR

The quantitative real-time PCR was conducted by use of method as described previously 25. The primers for tested genes were: GAPDH, Forward: 5′‐GGT GGT CTC CTC TGA CTT CAA CA‐3′, Reverse: 5′‐GTG GTC GTT GAG GGC AAT G‐3′; Interleukin-6 (IL-6), forward: 5'- ACT CAC CTC TTC AGA ACG AAT TG -3', reverse: 5'- CCA TCT TTG GAA GGT TCA GGT TG -3'; IL-10, forward: 5'- TCT CCG AGA TGC CTT CAG CAG A -3', reverse: 5'- TCA GAC AAG GCT TGG CAA CCC A -3'; TGF-β, forward: 5′- GGC CAG ATC CTG TCC AAG C -3′ and reverse: 5′- GTG GGT TTC CAC CAT TAG CAC -3′; tumor necrosis factor-alpha (TNF-α), forward: 5′- CCT CTC TCT AAT CAG CCC TCT G -3′ and reverse: 5′- GAG GAC CTG GGA GTA GAT GAG -3′; Nodal, forward: 5′- CTG CTT AGA GCG GTT TCA GAT G -3′ and reverse: 5′- CGA GAG GTT GGA GTA GAG CAT AA -3′. The primers of ABC transporters were described previously 26. The GAPDH was used as the internal control for normalization. The relative expression as calculated using the 2^-ΔΔCt^ method.

### Enzyme linked immunosorbent assay (ELISA)

The expression of Nodal in culture medium was tested by use of human Nodal ELISA kit (Invitrogen) according to manufactures' protocol and previous studies 27, 28.

### Cell transfection

The siRNA negative control (si-NC) or siRNA for Nodal were purchased from Genepharma (Genepharma, Shanghai, China). The sequence of si-NC was 5'-UUC UCC GAA CGU GUC ACG UTT-3', of si-Nodal was 5'-AGA TGG ACC TAT TCT CGA GAA T-3'. All transfection was conducted by use of Lipofectamine 2000 (Invitrogen, Carlsbad, CA, USA) according to the manufacturer's instructions.

### Western blot analysis

Proteins were extracted with Laemmli sample buffer and quantified by bicinchoninic acid assay (BCA). After separated by sodium dodecyl sulphate-polyacrylamide gel electrophoresis (SDS-PAGE, 6-12% gradient), proteins were transferred to polyvinylidene fluoride membranes (EMD Millipore), blocked with 5% skim milk and then incubated with primary antibodies at 4 °C overnight. The antibodies used in the present study were: Nodal (Abcam, ab110162, 1:500), P-gp (Abcam, ab129450, 1:500), p-p65 (p-S529-p65, Abcam, ab97726, 1:500), p65 (Abcam, ab16502, 1:500), and GAPDH (BOSTER, BM3876, 1:1000). After washed, membranes were incubated in secondary antibodies at room temperature for 1.5 h. Proteins were detected using enhanced chemiluminescent reagents (Thermo Fisher Scientific, Inc.) and visualized on X-ray film (Kodak, Japan). GAPDH was used as a loading control.

### Luciferase reporter assay

The promoter regions (-2000 to 0) of ABCB1 or Nodal were cloned into the pGL6-luc plasmid to generate promoter luciferase plasmids. Then cells were transfected with pGL6-luc and pRLTK (a plasmid containing the renila luciferase reporter gene) and further treated as the indicated conditions. The luciferase assay was conducted by use of the Dual-Luciferase® Reporter Assay System (Cat: E1910, Promega, USA). The results were calculated as relative light units/Renilla (pLR-TK).

### mRNA stability assay

Cells were treated with 5 μg/ml actinomycin D (Act-D, Catalogue #A9415, Sigma, St. Louis, MO, USA) to inhibit transcription. At each time point, cells were harvested and RNA was collected for qPCR analysis of ABCB1.

### Chromatin immunoprecipitation (ChIP)

The binding between transcription factors and Nodal promoter was checked by ChIP assay by use of Magna ChIP G Assay Kit (Millipore, Hayward, CA, USA) according to the manufacturer's instructions. Both parental and resistant cells were cross-linked with 37% formaldehyde, pelleted, and resuspended in lysis buffer. After sonication and centrifuge, the chromatin fragments were immunoprecipitated with antibodies and Protein Gmagnetic beads at 4 °C overnight. After dissociation at 62 ºC for 2 h, the precipitated DNA fragments were quantified using RT-PCR analysis. The primers for Nodal promoter were as follows: forward: 5' TCC CTC CAG GAT GTC TCG AGA GGC 3', reverse: 5' TTC AGG ATC CGC CAG CCC ACC ATG 3'.

### Statistical analysis

All experiments were performed three times independently and the average was used for comparison using the GraphPad Prism 5 software (GraphPad, USA). Two-tailed paired Student's t-tests and one-way ANOVA were used to compare different groups. P < 0.05 was considered as significant difference. * P < 0.05; ** P < 0.01, NS, no significant.

## Results

### The establish of CDDP resistant RCC cells

**Figure 1:**
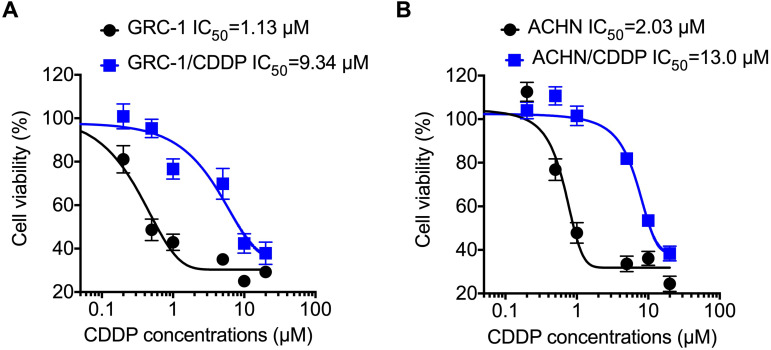
** The establish of RCC/CDDP cells**. GRC-1/CDDP (A), ACHN/CDDP (B) and their parental cells were treated with increasing concentrations of CDDP for 48 h, the cell viability was tested.

To understand molecular mechanisms underline chemoresistance, we established CDDP resistant RCC cells via treating cells with increasing concentrations of CDDP as described in methods. Cell viability assay showed that the RCC/CDDP cells were more resistant to that of RCC cells. The IC_50_ values of GRC-1/CDDP and GRC-1 were 9.34 and 1.13 μM, respectively (Figure 1A). The IC_50_ values of ACHN/CDDP and ACHN were 13.0 and 2.03 μM, respectively (Figure 1B).

### Nodal was increased in RCC/CDDP cells

**Figure 2:**
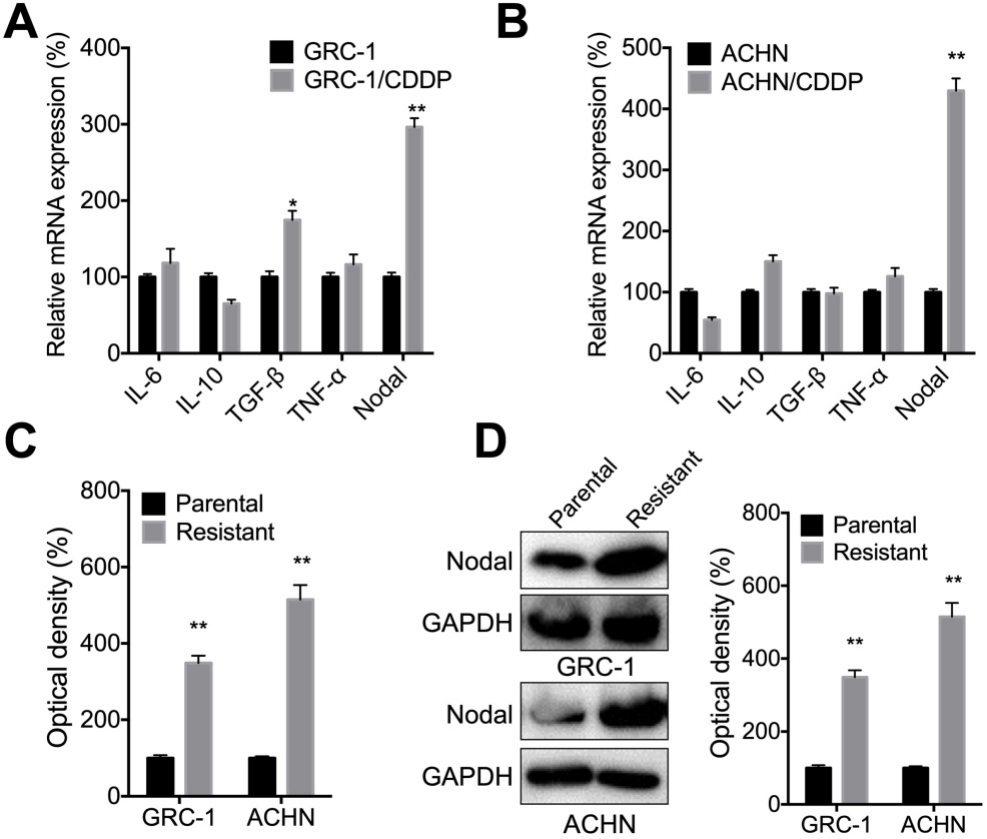
***Nodal was increased in RCC/CDDP cells***. The mRNA levels of measured cytokines in GRC-1/CDDP (A) and ACHN/CDDP (B) cells as compared to their corresponding control cells; The relative levels of Nodal in medium (C) or intracellular (D) of RCC/CDDP and RCC cells were measured by ELISA and western blot analysis, respectively.

Several cytokines such as IL-6, IL-10, TGF-β, TNF-α, and Nodal can regulate the chemoresistance of cancer cells 29. qRT-PCR showed that Nodal, while not IL-6, IL-10, TGF-β, or TNF-α, was increased in both GRC-1/CDDP (Figure 2A) and ACHN/CDDP (Figure 2B) cells as compared to their corresponding control cells. ELISA confirmed that levels of Nodal were increased in culture medium of both GRC-1/CDDP and ACHN/CDDP cells as compared to that in control cells (Figure 2C). Consistently, western blot analysis showed cellular levels of Nodal were also increased in RCC/CDDP cells (Figure 2D). All these results indicated that Nodal was increased in RCC/CDDP cells.

### Nodal regulated CDDP resistance of RCC cells

**Figure 3:**
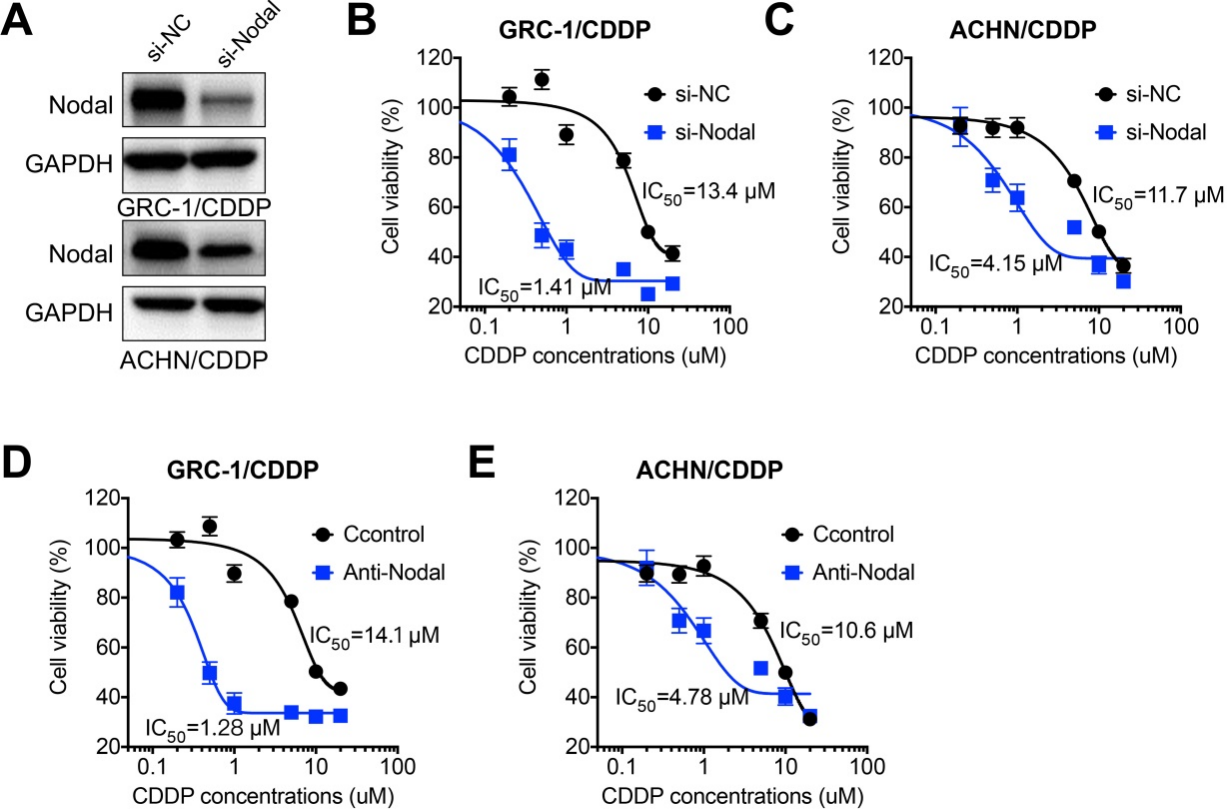
***Nodal regulated CDDP resistance of RCC cells***. (A) Cells were transfected with si-NC or si-Nodal for 24 h, the expression of Nodal was tested by western blot analysis; GRC-1/CDDP (B) or ACHN/CDDP (C) cells were pre-transfected with si-NC or si-Nodal for 24 h and then further treated with increasing concentrations of CDDP for 48 h; GRC-1/CDDP (D) or ACHN/CDDP (E) cells were pre-treated with or without anti-Nodal (100 ng/ml) for 12 h and then further treated with increasing concentrations of CDDP for 48 h.

In order to investigate whether Nodal was involved in chemoresistance of RCC cells, we knocked down the expression of Nodal via siRNA in both GRC-1/CDDP and ACHN/CDDP cells (Figure 3A). Our data showed that si-Nodal can significant restore CDDP sensitivity of both GRC-1/CDDP (Figure 3B) and ACHN/CDDP (Figure 3C) cells. Consistently, the neutralization antibody of Nodal (Anti-Nodal) can also increase CDDP sensitivity of both GRC-1/CDDP (Figure 3D) and ACHN/CDDP (Figure 3E) cells. It suggested that Nodal regulated CDDP resistance of RCC cells.

### Nodal regulated the expression of P-gp in RCC/CDDP cells

**Figure 4:**
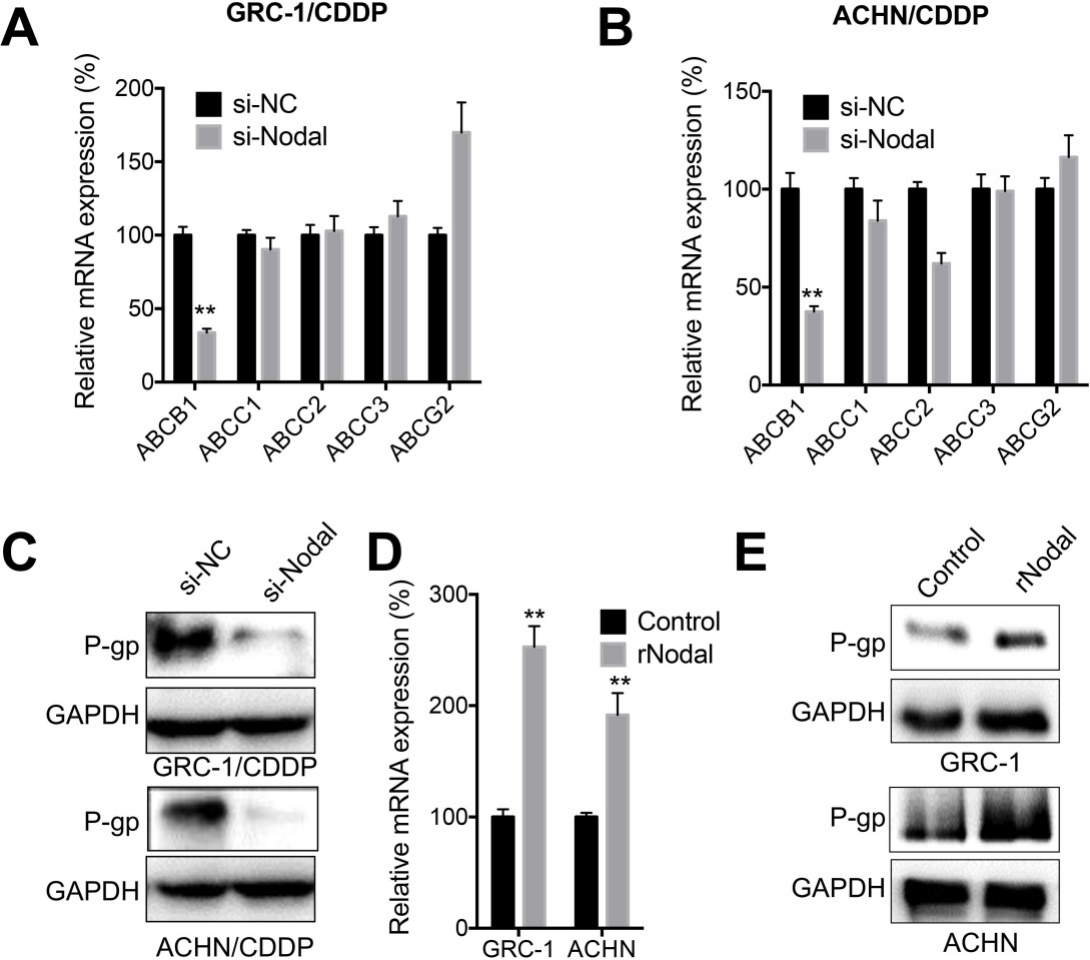
***Nodal regulated expression of P-gp in RCC/CDDP cells***. GRC-1/CDDP (A) or ACHN/CDDP (B) cells were transfected with si-NC or si-Nodal for 24 h, the mRNA expression of ABC transporters was checked; (C) The expression of P-gp in cells transfected with si-NC or si-Nodal for 24 h were tested; GRC-1 or ACHN cells were treated with or without rNodal (100 ng/ml) for 24 h, the mRNA (D) and protein (E) expression of P-gp was checked.

ABC family members including ABCB1, ABCC1, ABCC2, ABCC3, and ABCG2 were critical for CDDP resistance of cancer cells 30. Our data showed that knockdown of Nodal can significantly decrease expression of ABCB1 in both GRC-1/CDDP (Figure 4A) and ACHN/CDDP (Figure 4B) cells. Consistently, knockdown of Nodal also decreased protein expression of P-gp in RCC/CDDP cells (Figure 4C). In addition, both GRC-1 and ACHN cells treated with recombinant Nodal (rNodal) can increase mRNA (Figure 4D) and protein levels (Figure 4E) of P-gp. All these data indicated that Nodal can regulate expression of P-gp in RCC/CDDP cells.

### Nodal regulated the promoter activity of ABCB1 in RCC/CDDP cells

**Figure 5:**
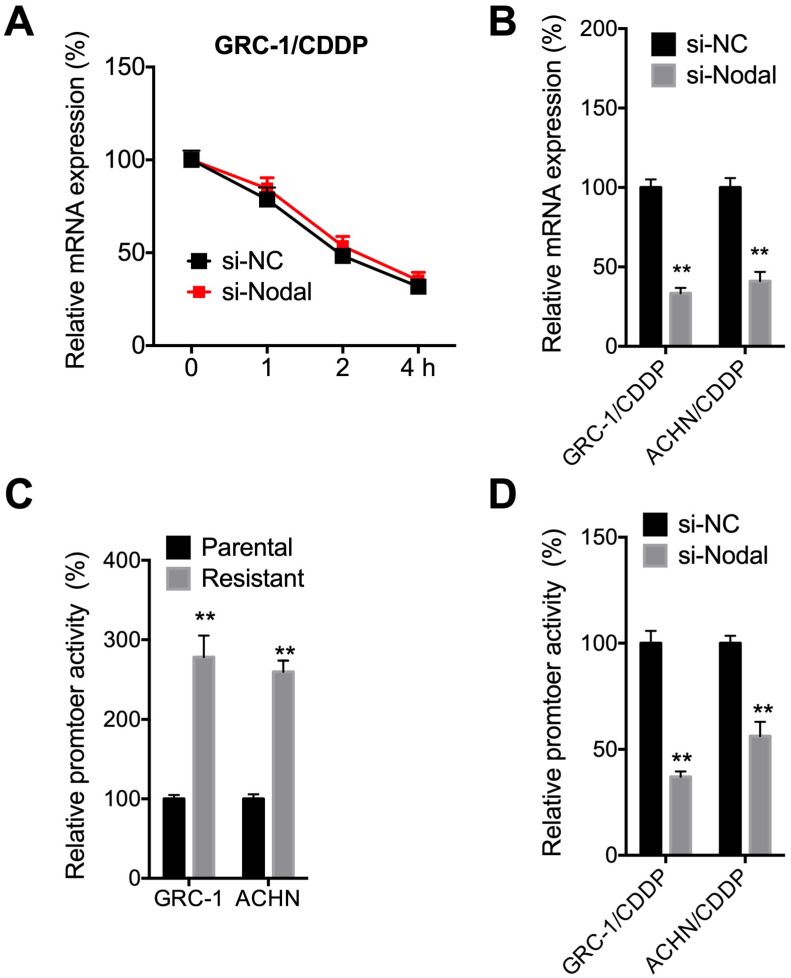
***Nodal regulated promoter activity of ABCB1 in RCC/CDDP cells***. (A) GRC-1/CDDP cells were pre-transfected with si-NC or si-Nodal for 24 h and then further treated with Act-D for the indicated times periods, the mRNA stability of ABCB1 was checked; (B) Cells were transfected with si-NC or si-Nodal for 24 h and precursor mRNA of ABCB1 was checked; (C) Both parental and resistant RCC cells were transfected pGL-ABCB1 and pRL-TK for 24 h, the luciferase activities of promoter were measured by dual-luciferase assay; (D) Cell pre-transfected with si-NC or si-Nodal were further transfected pGL-ABCB1 and pRL-TK for 24 h, the luciferase activities of promoter were measured.

We then investigated mechanisms responsible for Nodal induced expression of P-gp. The mRNA stability was tested since si-Nodal can decrease mRNA of ABCB1. Results showed that si-Nodal had no effect on mRNA stability of ABCB1 in GRC-1/CDDP cells (Figure 5A). However, si-Nodal can significantly decrease precursor-mRNA levels of ABCB1 in both GRC-1/CDDP and ACHN/CDDP cells (Figure 5B), which indicated that Nodal may regulate the transcription of ABCB1. We further tested the promoter activity of ABCB1 in RCC/CDDP cells and their control cells. Results showed that promoter activity of ABCB1 in both GRC-1/CDDP and ACHN/CDDP cells were greater than that in their corresponding control cells (Figure 5C). Further, si-Nodal can significantly decrease the promoter activity of ABCB1 in both GRC-1/CDDP and ACHN/CDDP cells (Figure 5D).

### NF-κB/p65 was involved in Nodal-regulated P-gp expression

**Figure 6:**
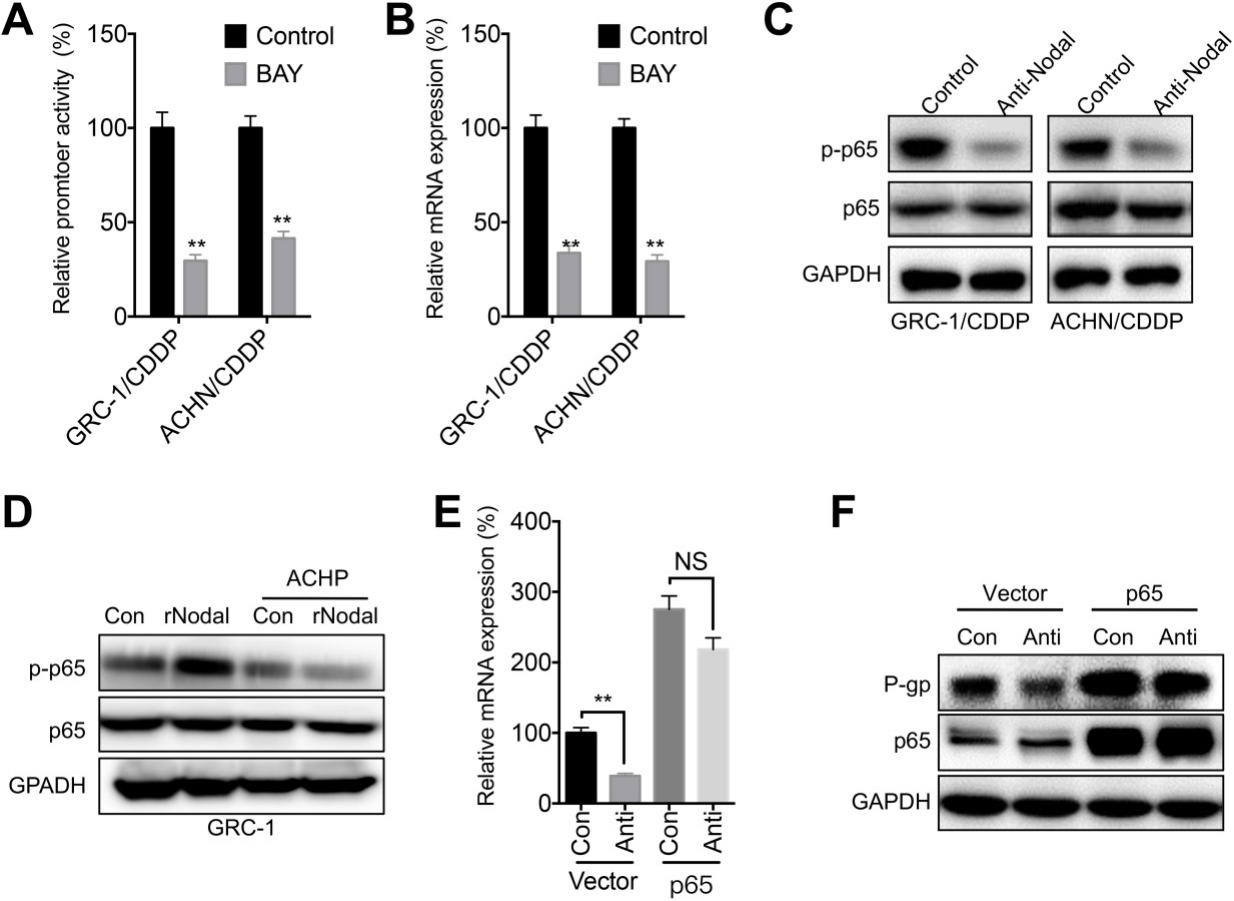
** NF-κB/p65 was involved in Nodal-regulated P-gp expression**. (A) Cells pre- transfected pGL-ABCB1 and pRL-TK for 12 h were further treated with or without BAY (10 µM) for 24 h, the promoter activity of ABCB1 was checked by luciferase assay; (B) Cells were treated with or without BAY (10 µM) for 24 h, the mRNA expression of ABCB1 was checked; (C) Cells were treated with or without anti-Nodal for 30 min, the phosphorylation and total expression of p65 were checked; (D) GRC-1 cells were pre-treated with control or ACHP (10 µM) for 90 min and then treated with rNodal (100 ng/ml) for 30 min. p-p65 and p65 were measured by western blot analysis; GRC-1/CDDP cells were pre-transfected with vector control (pcDNA3.1) or pcDNA/p65 for 12 h and then further treated with or without anti-Nodal for 24 h, the mRNA (E) or protein (F) levels of P-gp were measured.

NF-κB/p65 is one of the most frequently reported transcription factors regulating *ABCB1* expression 21. Our data showed that BAY 11-7082, the inhibitor of NF-κB, can significantly decrease promoter activity (Figure 6A) and mRNA expression (Figure 6B) of p65 in both GRC-1/CDDP and ACHN/CDDP cells. Further, anti-Nodal can significantly decrease the phosphorylation of p65 in both GRC-1/CDDP and ACHN/CDDP cells (Figure 6C). It has been showed that Nodal can increase phosphorylation of IKKβ/IκBα to activate NF-κB/p65 31. Our data also showed that ACHP, the inhibitor of IKK-β, can block rNodal induced phosphorylation of p65 in GRC-1 cells (Figure 6D). To verity the roles of p65 in Nodal-regulated transcription of ABCB1, cells were transfected with p65 constructs. Our data showed that over expression of p65 can attenuate anti-Nodal suppressed mRNA (Figure 6E) and protein (Figure 6F) expression of P-gp in GRC-1/CDDP cells.

### CDDP treatment increased Nodal expression in RCC cells

**Figure 7:**
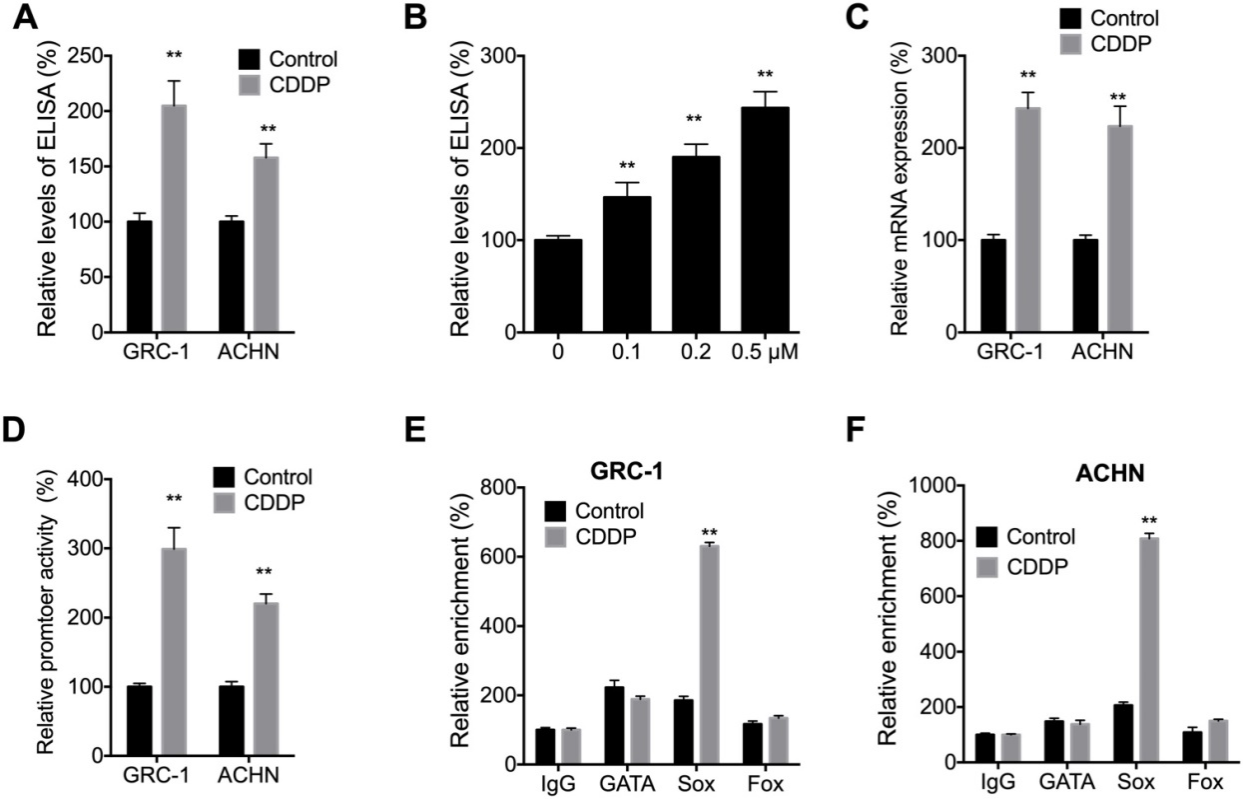
** CDDP increased Nodal expression in RCC cells**. (A) Cells were treated with or without 0.2 µM CDDP for 24 h, the expression of Nodal in medium was checked; (B) GRC-1 cells were treated with increasing concentrations of CDDP for 24 h, the expression of Nodal in medium was checked; Cells were treated with or without 0.2 µM CDDP for 24 h, the mRNA expression (C) or promoter activity (D) of Nodal were checked; GRC-1 (E) or ACHN (D) cells were treated with or without 0.2 µM CDDP for 24 h, the binding of Nodal promoter and transcription factors were checked by ChIP-PCR.

We further investigated the potential effects of CDDP on Nodal expression in RCC cells. ELISA showed that CDDP can increases the levels of Nodal in both GRC-1 and ACHN cells (Figure 7A). In addition, CDDP can increase levels of Nodal via a concentration dependent manner in GRC-1 cells (Figure 7B). Consistently, CDDP can increase mRNA levels of Nodal in both GRC-1 and ACHN cells (Figure 7C). It might be due to that CDDP can increase promoter activity of Nodal in RCC cells (Figure 7D). The data indicated that CDDP treatment can increase Nodal expression in RCC cells.

It has been revealed that the expression of Nodal can be regulated by expression of GATA, Sox, and Fox transcription factors 32. We then checked the binding between GATA, Sox, and Fox transcription factors with the promoter of Nodal in cells treated with or without CDDP. Our data showed that CDDP treatment can increase the binding between Sox and Nodal promoter in both GRC-1 and ACHN cells (Figure 7E and F). It suggested that Sox might be involved in CDDP induced transcription of Nodal. However, mechanisms responsible for CDDP induced Sox activation need further investigation.

## Discussion

Previous studies indicated that TGF-β signal pathway is critical for the development and progression of RCC 33, 34. Our present study revealed that Nodal, one member of TGF-β family, can mediate the CDDP chemoresistance of RCC cells. It was evidenced by results that Nodal was increased in RCC/CDDP cells, while block the Nodal functions via its siRNA or neutralization antibody can restore CDDP sensitivity of RCC cells. As an embryonic morphogen, Nodal can re-express in cancer cells to increase the aggressiveness and tumorigenicity of various cancers 35, 36. For example, Nodal can increase migration of prostate 37 and breast cancer 38 cells. As to chemoresistance, it has been reported that Nodal induced by hypoxia exposure contributes to dacarbazine resistance in melanoma cancer stem‑like cells 22. Our data, together with published results, suggested that Nodal is also involved in chemoresistance of cancer cells and might be a target for overcome chemoresistance of RCC.

Our data suggested that P-gp is essential for Nodal induced CDDP resistance of RCC cells. P-gp has been well known as the chemotherapy drug transporter in various chemoresistant cancer cells 39. Our data found that Nodal can regulate the expression of ABCB1, while had no effect on other measured transporters. Mechanistically, Nodal can increase the transcription of ABCB1 in RCC cells, which was evidenced by the results that Nodal can regulate promoter activity and precursor mRNA of ABCB1 in RCC cells. It has been revealed that P-gp is a possible adverse prognostic factor of chemoresistance and aggressive behaviour in RCC cells 12. In addition, longer disease-free survival was observed in patients with lower levels of P-gp in RCC patients 40. All these observations confirmed that P-gp might be one important effector for Nodal-induced malignancy of RCC.

NF-κB/p65 activation is involved in Nodal induced expression of ABCB1 in RCC cells, which was evidenced by results that Nodal can increase phosphorylation of p65, while over expression of p65 can attenuate si-Nodal-suppressed transcription of ABCB1. There are several NF-κB binding sties in human ABCB1 promoter 41, 42, further, NF-κB and other transcription factors such as AP-1 and SP-1 can bind with ABCB1 promoter to increase its transcription 43. In lung cancer cells, Nodal can promote the malignancy of cancer cells via activation of NF-κB/IL-6 signal pathways 31. The p65 can be activated via IKK/ IκBα signals in cancer cells 44. It has been revealed that specific pro-inflammatory signals such as TNF-α and IL-1β can phosphorylate IκBα and trigger its degradation, which results in a rapid nuclear translocation and thereby activation of NF-κB 45. Whether IKK/ IκBα was involved in Nodal-induced activation of p65 need further study. In addition, posttranslational modifications like HDAC mediated acetylation or deacetylation can also regulate the activation of p65 46. Whether the mentioned factors and other factors such as AP-1 and SP-1 are also involved in Nodal induced P-gp expression needs further studies.

Collectively, our study revealed that Nodal can regulate chemoresistance of RCC cells via modulation of P-gp expression. The activation of p65 is involved in Nodal regulated P-gp expression and chemoresistance. Although clinical evidences are required to confirm the roles of Nodal on RCC progression, our present study suggested that Nodal might be a target for overcome the chemoresistance.
